# College and Hospital Notes

**Published:** 1906-01

**Authors:** 


### COLLEGE AND HOSPITAL NOTES.

---

THE New York Post-Graduate College and Hospital recently issued its annual report which covers the year ending October 1, 1905, and which is the twenty-first annual report of the directors. Dr. D. B. St. John Roosa is the president of the corporation as well as of the faculty, Dr. Andrew H. Smith is the vice-president. Dr. Bache McE. Emmet is the treasurer and Dr. George N. Miller is the secretary. The members of the corporation constitute a strong body of men, some of whom are among the most influential citizens of New York. The report makes a brochure of about 110 duodecimo pages and is a very handsome book. It is well illustrated with exterior and interior views of the buildings of the corporation. One full page view shows 21 of as comely nurses as we ever saw in a group and the entire report is a credit to the institution.

---

THE Buffalo Emergency Hospital staff at its annual meeting elected the following officers: president, Dr. Francis J. Carr; vice-president, Dr. Vertner Kenerson; secretary, Dr. Edgar A. Forsyth.

---

THE Buffalo Hospital of the Sisters of Charity held its annual meeting recently at which the following officers were elected: president, Dr. George Westinghouse; vice-president, Dr. Walter D. Greene; secretary, Dr. Alfred E. Diehl.

